# The characteristics of oro-cervical necrotizing fasciitis—Comparison with severe cellulitis of oro-cervical region and necrotizing fasciitis of other body regions

**DOI:** 10.1371/journal.pone.0260740

**Published:** 2021-12-01

**Authors:** Eiji Iwata, Junya Kusumoto, Naoki Takata, Shungo Furudoi, Akira Tachibana, Masaya Akashi

**Affiliations:** 1 Department of Oral and Maxillofacial Surgery, Kakogawa Central City Hospital, Kakogawa, Japan; 2 Department of Oral and Maxillofacial Surgery, Kobe University Graduate School of Medicine, Kobe, Japan; 3 Department of Oral and Maxillofacial Surgery, Konan Medical Center, Kobe, Japan; Mayo Clinic Rochester: Mayo Clinic Minnesota, UNITED STATES

## Abstract

**Background:**

Necrotizing fasciitis (NF) is an acute and life-threatening soft-tissue infection however rarely seen in oro-cervical region. Therefore, the details of oro-cervical NF (OCNF) are not well known. The purpose of this study was to investigate the characteristics of OCNF by comparing it with severe cellulitis of oro-cervical region (OCSC) or NF of other body regions (e.g., limb, perineum, and trunk) (BNF), respectively.

**Materials and methods:**

At first, various risk factors for OCNF in oro-cervical severe infection (OCSI; composed of OCNF and OCSC), including neutrophil-to-lymphocyte ratio (NLR) and Laboratory Risk Indicator for Necrotizing Fasciitis (LRINEC) score, were investigated by univariate and multivariate analyses. Next, the differences between OCNF and BNF, including inflammatory markers and mortality, were investigated.

**Results:**

In the present study, 14 out of 231 OCSI patients had OCNF. Multivariate analyses of OCSI patients showed that NLR ≥15.3 and LRINEC score ≥6 points were significantly related to OCNF. During the same period, 17 patients had BNF. The OCNF group had significantly higher inflammatory markers than the BNF group when diagnosis, but significantly lower clinical stages at the time and mortality as outcomes.

**Conclusion:**

We found that compared to BNF, OCNF can be detected at lower clinical stage by using indexes, such as NLR and LRINEC score, besides clinical findings, which may help contributing to patient’s relief.

## 1. Introduction

Necrotizing fasciitis (NF) is a severe form of infection involving rapidly spreading inflammation and extensive necrosis of the skin, subcutaneous tissue, and superficial- fascia [1, 2]. If diagnosis and surgical treatment are delayed, NF can lead to systemic toxicity, multisystem organ failure, and eventual death [3–5]. In the past, many studies have investigated the characteristics of NF of the limb, perineum, and trunk, such as prognosis and severity [6–12]. Therefore, many surgeons are aware of the characteristics of NF in these regions.

On the other hand, NF is rarely seen in oro-cervical region (OCNF). Sepulveda and Sastre reported that the incidence of OCNF is less likely because of the abundant blood supply in the oro-cervical region [13]. However, the spread of oro-cervical infection creates a risk of asphyxiation and because the retropharyngeal space and the spaces, adjacent to the oro-cervical region are continuous with the mediastinum, delayed diagnosis and treatment may lead to fatal conditions, such as mediastinitis. Unfortunately, because of its low incidence, many surgeons are unfamiliar with this condition.

In the present study, we compared the characteristics of OCNF with those of severe cellulitis of oro-cervical region (OCSC) and NF of other body regions (e.g., limb, perineum, and trunk) (BNF).

## 2. Patients and methods

### 2.1. Patients

In this study, the following inclusion criteria were set: patients above 18 years of age, and patients who were hospitalized for the treatment of OCSC with drip antibiotics for over 48 hours. The following exclusion criteria were set: patients with cancer, patients who were transferred to other hospitals for some reasons, patients who did not wish to participate after the publication of this study, and patients with missing data which needed in this study (the detail is described in 2.4. “Variables”). We examined patients who were treated for oro-cervical severe infection (OCSI; composed of OCSC and OCNF) or BNF between April 2012 and March 2020 in Kakogawa Central City Hospital. This study was performed in accordance with the 1964 Declaration of Helsinki. Ethical approval was obtained from the Institutional Review Boards (IRB) of Kakogawa Central City Hospital (Authorization number: 2019–85). The ethics committee approved gave us administrative permissions to access the data used in this study. As this was a retrospective study, the research plan was published on the homepage of the hospital according to the instructions of the IRB in accordance with the guaranteed opt-out opportunity.

### 2.2. Necrotizing fasciitis (NF)

Patients corresponding with Fisher et al. ‘s definition of NF [14] and Mathieu et al. ‘s definition of CNF [15] were defined as NF. Fisher et al. [14] defined the following features: (1) necrosis of extensive superficial-fascia and surrounding tissues, (2) moderate or severe systemic intoxication with psychiatric symptoms, (3) muscle layer is not affected, (4) clostridium is not detected in wounds and blood, (5) no obstruction of large vessels, (6) severe leukocyte infiltration, fascia and surrounding tissue necrosis, and microvascular thrombosis observed on histopathological examination. Mathieu et al. [15] defined the following features: (1) inflammation in the submandibular space, with little or no pus and with spread to the neck beyond the level of the hyoid bone; (2) involvement of more than one neck space, usually bilaterally; (3) tissue necrosis with serosanguineous, putrid infiltration; (4) involvement of connective tissue and fasciae and, in a secondary manner, muscles and skin, but not of glandular structures; and (5) contiguous―not lymphatic―spread. Even if all items of Fisher et al. ‘s definition and Mathieu et al. ‘s definition were not satisfied, NF was finally defined when the case in which fascial necrosis was confirmed by intraoperative findings and histopathology.

NF is broadly classified into type 1, which is a polymicrobial infection containing anaerobes (e.g., *Bacteroides fragilis*, *Prevotella spp*., *Escherichia Coli*), and type 2 which is a monomicrobial infection (e.g., *Streptococcus pyogenes*, *Staphylococcus aureus*) [16–18]. NF does not originally produce gas; however, some anaerobes, which are the causative bacteria of type 1 NF, produce gas. Since type 1 NF has almost the same disease state as non-Clostridium gas gangrene, NF with gas production is almost synonymous with non-Clostridium gas gangrene [19]. Therefore, in the present study, patients in whom gas production was observed on CT images were also included in the NF for the above reasons.

### 2.3. Severe cellulitis of oro-cervical region (OCSC)

The following patients were defined as OCSC: patients with clinical findings in the oro-cervical region, such as erythema, swelling, and heat, who had difficulty eating or breathing, and who were hospitalized for treatment with intravenous antibiotics for over 48 hours. Patients diagnosed with OCNF were excluded.

### 2.4. Variables

The following variables from medical records were retrospectively reviewed and investigated. All exams including blood test and radiographic test were done at the time of admission. (1) patient factors―sex, age, body mass index (BMI), and compromised host; (2) clinical findings factors—creatine kinase (CK), C-reactive protein (CRP), white blood cell count (WBC), neutrophil-to-lymphocyte ratio (NLR), hemoglobin (Hb), serum creatinine (Cr), sodium (Na), blood glucose (Glu), and LRINEC score; and (3) treatment progress―hospitalization periods and outcomes. Moreover, the cases of NF were additionally investigated for 2 clinical findings; image in computed tomography (CT), and clinical stages.

In patient factors, “compromised host” was defined as a patient with the following diseases; e.g., rheumatoid arthritis, kidney failure, osteoporosis, and diabetes. In clinical finding factors, CK, Hb, and Cr have different normal values by sex. We valued them by sex. In addition, we used 2 risk scores and 1 clinical stage. NLR and LRINEC score, both developed to predict the severity and prognosis of NF, such as Fournier’s Gangrene [20, 21]. LRINEC score is a clinical tool first described by Wong et al. The tool is based on 6 common serum parameters: CRP, WBC, Hb, Cr and Glu (Table 1). Most of BNF reports set LRINEC score cutoff value at 6 points [22–26]. Whereas, few reports investigated whether LRINEC score is useful index of diagnosing OCNF [27, 28]. Namely, the appropriate cutoff value is unknown unlike BNF. In this study, the cutoff values for NLR and LRINEC score in OCSI patients were determined using receiver operating characteristic (ROC) curve analysis. “Clinical stages” were used in a study by Wong and Wang [29] (Table 2). It is classified into 3 stages based on changes in the skin as the disease progresses (Table 2). Finally, regarding the presence of gas production images in CT, the cases in which the abscess self-collapsed and cases in which gas was continuous with the outside in CT were excluded because they are likely to be air bubbles.

**Table 1:** LRINEC score.

Variable	Value	Points
CRP (mg/dL)	≥15	4
WBC (/μL)	≥15,000	1
	>25,000	2
Hb (g/dL)	<13.5	1
	<11.0	2
Na (mmol/dL)	<135	2
Cr (mg/dL)	>1.59	2
Glu (mg/dL)	>180	1

**Table 2:** Clinical features of NF as the disease progress through clinical stages.

Stage 1 (Early)	Stage 2 (intermediate)	Stage 3 (Late)
Tenderness to palpation (extending beyond the apparent area of skin involvement)	Blister or bullae formation (serious fluid)	Hemorrhagic bullae
		Skin anesthesia
Erythema	Skin fluctuance	Crepitus
Swelling	Skin induration	Skin necrosis with dusky discoloration progressing to frank gangrene
Warm to palpation		

### 2.5. Statistical analyses

Statistical analyses were performed using SPSS 26.0 (SPSS, Chicago, IL). The association of each variable with OCNF, in each between OCNF group and OCSC group and between OCNF group and BNF group, was analyzed by the Mann-Whitney U nonparametric test for ordinal variables and Fisher’s exact test or the chi-square test for categorical variables. Probabilities of less than 0.05 were accepted as significant. All of the variables associated with OCNF in OCSI patients were introduced into a multiple logistic regression model. Before introduction multicollinearity test was done, with the rejection of those variables that did not fit the model significantly. Moreover, after multiple logistic regression, goodness-of-fit analysis was done. About presence or absence of multicollinearity between each factor, Variance Inflation Factor (VIF) was used. As a goodness-of-fit analysis, Hosmer and Lemeshow Test was used. OR and 95% confidence intervals (CIs) were also calculated.

## 3. Results

### 3.1. Comparison of OCNF with OCSC

**Fig 1:**
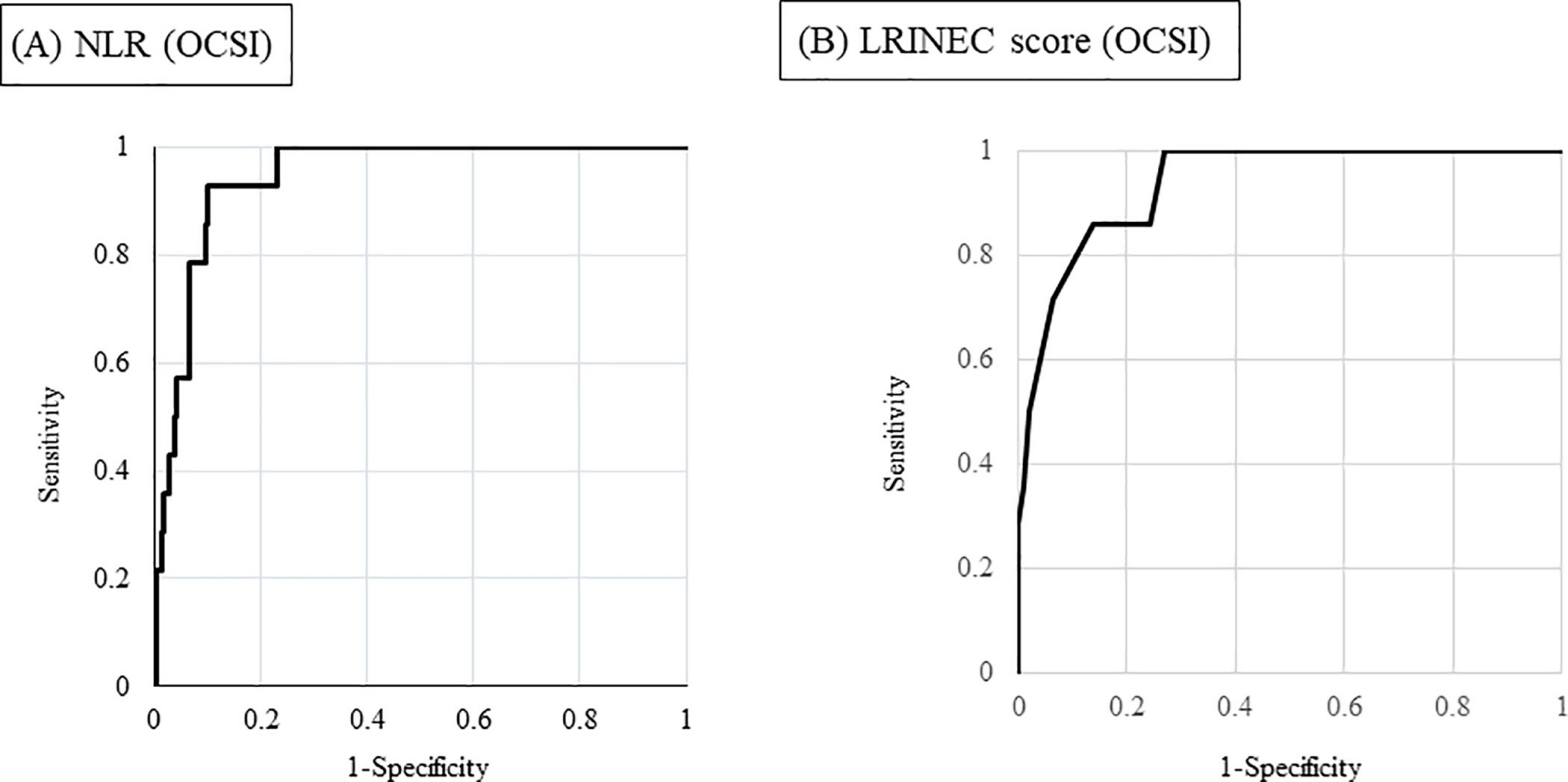
(A) The ROC curve for accuracy of NLR in predicting the presence of OCNF in OCSI patients. The AUC for our model was 0.944 (95% confidence interval 0.907 to 0.982). (B) The ROC curve for accuracy of LRINEC score in predicting the presence of OCNF in OCSI patients. The AUC for our model was 0.938 (95% confidence interval 0.889 to 0.987).

OCNF occurred in 14 of 231 patients with OCSI (6.1%) (Table 3). The remaining 219 patients were defined as having OCSC. NLR ≥15.3 had a sensitivity of 92.9%, a specificity of 90.0%, and area under curve (AUC) of 0.94 (Fig 1A). LRINEC score of ≥6 points had a sensitivity of 71.4%, a specificity of 93.5%, and an AUC of 0.94 (Fig 1B). We set these as cutoff values of NLR or LRINEC score, respectively. 8 OCNF patients and 57 OCSC patients were diagnosed as the compromised host (Table 3). 8 OCNF patients had osteoporosis (3 patients), diabetes (2 patients), kidney failure (2 patient), and collagen disease using steroid (1 patient). 57 OCSC patients consisted of diabetes (23 patients*), osteoporosis (16 patients*), kidney failure (10 patient*), and collagen disease using steroid (7 patient*) (*there is a duplication).

**Table 3:** Results of univariate analysis of the risk factors for OCNF in OCSI patients.

Variables		OCNF group	OCSC group	*P* value
		(n = 14)	(n = 217)	
Sex	Male	5 (35.7)	110 (50.7)	0.409 α
	Female	9 (64.3)	107 (49.3)	
Age	Mean ± SD	69.1 ± 17.3	56.0± 21.2	0.022* β
BMI	Mean ± SD	21.9 ± 3.6	22.9 ± 4.0	0.349 β
Compromised host	No	6 (42.9)	160 (73.7)	0.026* α
	Yes	8 (57.1)	57 (26.3)	
CK (U/L)	Mean ± SD	247.3 ± 608.2	115.1 ± 178.7	0.599 β
Male	Mean ± SD	604.0 ± 976.3	137.6 ± 211.8	0.046* β
Female	Mean ± SD	49.1 ± 26.9	92.0 ± 133.8	0.102 β
CRP (mg/dL)	Mean ± SD	26.4 ± 10.2	10.0 ± 5.9	<0.001* β
WBC (10^3^/μL)	Mean ± SD	22.5 ± 10.6	12.4 ± 3.6	<0.001* β
Hb (g/dL)	Mean ± SD	11.7 ± 1.6	13.5 ± 1.8	0.001* β
Male	Mean ± SD	12.6 ± 1.4	14.3 ± 1.6	0.022* β
Female	Mean ± SD	11.2 ± 1.6	12.6 ± 1.6	0.022* β
Na (mmol/L)	Mean ± SD	137.2 ± 4.6	138.3 ± 5.1	0.394 β
Cr (mg/dL)	Mean ± SD	1.2 ± 0.6	0.9 ± 0.5	0.043* β
Male	Mean ± SD	1.3 ± 0.5	0.9 ± 0.4	0.019* β
Female	Mean ± SD	1.0 ± 0.7	0.9 ± 1.3	0.394 β
Glu (mg/dL)	Mean ± SD	138.6 ± 51.1	125.7 ± 45.6	0.066 β
NLR	<15.3 (cut off value)	1 (7.1)	195 (89.9)	<0.001* β
	≥15.3	13 (92.9)	22 (10.1)	
LRINEC score	<6 points (cut off value)	4 (28.6)	203 (93.5)	<0.001* α
	≥6 points	10 (71.4)	14 (6.5)	
Hospitalization periods (days)	Mean ± SD	24.3 ± 14.2	9.6 ± 6.3	<0.001* β
Outcomes	Survival	13 (92.3)	217 (100.0)	1.000 α
	Mortality	1 (7.7)	0 (0.0)	

Values are expressed as absolute numbers, with the corresponding percentage of the total in parentheses. Values in the right-hand column indicate the statistical significance of the difference between subgroups. Most variables expressed as the mean ± standard deviation in a parametric ratio scale.

α: Fisher’s exact test; β: Mann–Whitney U test; γ: Chi-squared test. * P < 0.05.

Age, compromised host, CK (male), CRP, WBC, Hb, Cr, NLR≥15.3, and LRINEC score≥6 points had a statistically significant correlation with OCNF according to the univariate analysis outcomes (Table 3). The OCNF group was hospitalized for significantly longer period than the OCSC group (Table 3). One of the 14 patients died due to OCNF (Table 3).

Before introduction into a multiple logistic regression model, multicollinearity test was done by VIF. As a result, the VIF value was as low as less than 3, and no multicollinearity was observed. Therefore, not any variables were excluded. Multivariate analysis showed that NLR≥15.3 (OR: 63.5, 95% CI: 7.4–545.7) and LRINEC score≥6 points (OR: 14.8, 95% CI: 3.1–69.7) were significant risk factors for OCNF in OCSI patients (Table 4). After multivariate analysis, the goodness-of-fit analysis by using Hosmer and Lemeshow Test was done. As a result, the value showed 0.317. It means that there was no problem with the goodness of fit of the model.

**Table 4:** The results of the multivariate logistic regression analysis of the risk factors for OCNF in OCSI patients.

			95% CI	
Variable	P value	Odds ratio	Lower	Upper
NLR ≥15.3	<0.001	63.5	7.4	545.7
LRINEC score ≥6 points	0.001	14.8	3.1	69.7

CI. Confidence interval.

Data are the p-value, odds ratio and 95% confidence interval (CI) for those factors found to be significantly associated with an increased risk of OCNF.

### 3.2. Comparison OCNF with BNF

NF occurred in 31 patients, of which 14 had OCNF and 17 had BNF (Table 5). The sites where NF occurred the most were mandible (92.3%) in the OCNF group and lower limbs (47.1%) in the BNF group, respectively (Table 5). 8 OCNF patients and 5 BNF patients were diagnosed as compromised host (Table 5). The detail of OCNF group was as mentioned above. 5 BNF patients had kidney failure (3 patients), diabetes (2 patients), and rheumatoid arthritis (1 patient).

**Table 5:** Comparison of OCNF group and BNF group.

Variables		OCNF group	BNF group	*P* value
		(n = 14)	(n = 17)	
Site of infection	Lower limb	―	8 (47.1)	― γ
	Upper limb	―	3 (17.6)	
	Perineum	―	5 (29.4)	
	Trunk	―	1 (5.9)	
	Maxilla	1 (7.7)	―	
	Mandible	13 (92.3)	―	
Sex	Male	5 (35.7)	9 (52.9)	0.473 α
	Female	9 (64.3)	8 (47.1)	
Age	Mean ± SD	69.1 ± 17.3	63.8 ± 18.4	0.444 β
BMI	Mean ± SD	21.9 ± 3.6	21.9 ± 2.3	0.830 β
Compromised host	No	6 (42.9)	12 (70.6)	0.157 α
	Yes	8 (57.1)	5 (29.4)	
CK (U/L)	Mean ± SD	247.3 ± 608.2	350.0 ± 638.8	0.336 β
Male	Mean ± SD	604.0 ± 976.3	176.1 ± 246.8	0.190 β
Female	Mean ± SD	49.1 ± 26.9	545.4 ± 883.5	0.059 β
CRP (mg/dL)	Mean ± SD	26.4 ± 10.2	18.8 ± 10.2	0.044* β
WBC (10^3^/μL)	Mean ± SD	22.5 ± 10.6	13.5 ± 6.6	0.021* β
Hb (g/dL)	Mean ± SD	11.7 ± 1.6	11.5 ± 2.2	0.739 β
Male	Mean ± SD	12.6 ± 1.4	11.3 ± 2.7	0.240 β
Female	Mean ± SD	11.2 ± 1.6	11.8 ± 1.7	0.370 β
Na (mmol/L)	Mean ± SD	137.2 ± 4.6	133.9 ± 5.3	0.071 β
Cr (mg/dL)	Mean ± SD	1.2 ± 0.6	2.4 ± 3.0	0.377 β
Male	Mean ± SD	1.3 ± 0.5	3.0 ± 3.7	0.898 β
Female	Mean ± SD	1.0 ± 0.7	1.8 ± 2.0	0.423 β
Glu (mg/dL)	Mean ± SD	138.6 ± 51.1	167.7 ± 134.5	0.625 β
NLR	Mean ± SD	26.0 ± 14.0	24.6 ± 18.6	0.468 β
LRINEC score	<6 points	4 (28.6)	7 (41.1)	0.423 α
	≥6 points	10 (71.4)	10 (58.9)	
Gas production	No	9 (64.3)	14 (82.4)	0.412 α
	Yes	5 (35.7)	3 (17.6)	
Clinical stages	Stage 1	14 (100.0)	6 (35.3)	<0.001* γ
	Stage 2	0 (0.0)	4 (23.5)	
	Stage 3	0 (0.0)	7 (41.2)	
Hospitalization periods (days)	Mean ± SD	24.3 ± 14.2	30.1 ± 19.9	0.376 β
Outcomes	Survival	13 (92.3)	11 (64.7)	0.073 α
	Mortality	1 (7.7)	6 (35.3)	

Values are expressed as absolute numbers, with the corresponding percentage of the total in parentheses. Values in the right-hand column indicate the statistical significance of the difference between subgroups. Most variables expressed as the mean ± standard deviation in a parametric ratio scale.

α: Fisher’s exact test; β: Mann–Whitney U test; γ: Chi-squared test.

* P < 0.05.

Univariate analyses showed that CRP, WBC, and clinical stages had a statistically significant correlation between the OCNF and BNF groups (Table 5). The OCNF group had significantly higher inflammatory marker levels than the BNF group, but significantly lower disease progression (Table 5). The hospitalization periods were almost the same among 2 groups; however, the OCNF group had much lower mortality than the BNF group (Table 5).

## 4. Discussion

In the present study, we investigated the characteristics of OCNF in comparison with OCSC and BNF. Multivariate analyses showed that NLR ≥15.3 and LRINEC score ≥6 points were significantly related to OCNF in OCSI patients. On the other hand, univariate analyses of NF showed that CRP, WBC and clinical stages were significant factors. Furthermore, the OCNF group had much lower mortality than the BNF group.

In the patients with OCSI, univariate analyses showed that age, compromised host, CK (male), CRP, WBC, Hb, Cr, NLR ≥15.3, and LRINEC score ≥6 points were significantly associated with OCNF. Simonart et al. reported that CK is important for the early diagnosis of BNF [30]. Several reports have shown that infection-driven inflammation correlates with markers of malnutrition and inflammation, such as CRP, WBC, Hb, and Cr in blood tests [31, 32]. When inflammation becomes severe, CRP, WBC, and Cr tend to increase, while Hb tends to decrease [31, 32]. In the present study, OCNF showed such a tendency significantly when compared with OCSC. Several reports have shown that LRINEC score is useful for predicting BNF [22–26]. Most of the reports set LRINEC score cutoff value at 6 points, which is similar to the present study. However, only 2 reports have investigated the usefulness of LRINEC score for predicting OCNF [27, 28]. Although both reports both set LRINEC score cutoff value at 6 points and the same definition of OCNF and OCSC, opinions on the usefulness of the score were exact opposite [27, 28]. This may be because both reports were quite different in the proportion of OCNF in OCSI patients (17 vs. 70 and 16 vs. 595) [27, 28]. In this study, the proportion was 14 vs. 217 and the definitions of OCNF and OCSC were also similar to previous studies [27, 28]. To our knowledge, no reports have investigated the relationship between NLR and NF. However, NLR is noticed as the useful index related to several diseases there days. Actually, several reports have shown that NLR can predict the outcomes of patients with cancers [33–37]. In the present study, we found that NLR is also useful for predicting OCNF. NLR is much easier calculation method than LRINEC score because of just rate dividing the neutrophil by lymphocyte.

In the patients with NF, the OCNF group had significantly higher inflammatory markers than the BNF group, but significantly lower clinical stages when diagnosed clinically. Furthermore, the OCNF group (7.7%) had much lower mortality than the BNF group (35.3%) These findings showed that when compared with BNF, OCNF can be detected at a lower clinical stage and therefore, the surgeons may be better prepared to save lives. This may be because, compared to BNF, OCNF is easily noticed at an earlier stage (in the state of OCSC or early stage of OCNF), by patients themselves or their family members, as a complication in the oro-cervical region, such as trismus, dysphagia, and skin flare. NF makes superficial-fascia with poor blood flow the main base of bacterial infection; however, several reports showed that because of the abundant blood supply in oro-cervical regions, OCNF is rare in comparison to BNF [13, 38, 39]. In addition, some reports have shown that the superficial cervical fascia is thinner than those of other parts including limbs and trunk [40, 41]. The thinness of the superficial cervical fascia may be associated with the patient’s increased awareness of symptoms and, consequently, the rareness of OCNF. In fact, OCNF is reported in 2.6–5.0% among all cases of NF [1, 42]. Next, we considered the difference between OCNF and BNF from the aspect of NF type. Type 1 NF which is 70–80% of NF, occurs in immunocompromised individuals, such as patients with diabetes mellitus or chronic renal failure, infection of the oro-cervical region, abdominal wall, and surgical wounds is common, and gas production can be sometimes seen [16–18]. Type 2 NF can be caused by trauma to the limbs (especially lower limbs) even in young healthy people, with a very high mortality rate of 40% when it occurs [16–18]. In the present study, the causative bacteria were identified in half of the OCNF group (7 out of 14 cases), in which multiple bacterial species including anaerobic bacteria, such as *Prevotella spp*. and *Peptostreptococcus spp*. Most of the OCNFs were probably type 1 NF because the cases in which causative bacteria were not detected were also accompanied by a strong anaerobic odor and anaerobic bacteria are actually difficult to culture. In the BNF group, the causative strain was identified in 14 out of 17 cases. Of these, 9 were *Streptococcus pyogenes*, 5 were multiple strains including *Bacteroides fragilis* and *Escherichia coli*. In other words, more than half of the BNFs were type 2 NFs. In fact, the OCNF group (57.1%) had a higher number of compromised host patients than the BNF group (29.4%). In addition, the OCNF group (35.7%) had much more gas production than the BNF group (17.6%). This may be because OCSI is a dental infection that spreads through "spaces" of the oro-cervical regions, unlike BNF. When the infection reaches the fascia (i.e., OCNF), the infection has already spread to the spaces, which may also have been accompanied by gas production due to some anaerobes. This infection route of OCNF, which is different from that of BNF, may cause severe local symptoms early and may have led to different results regardless of the same NF.

As a treatment of NF, early administering antibiotics and surgical debridement are needed to rescue the patients. Broad-spectrum antibiotics should be chosen, as the basic antibacterial therapy. Generally, double- or triple-combination is recommended as the basic criterion, including third-generation cephalosporins, aminoglycoside antibiotics, or metronidazole [43]. Furthermore, such as meropenem and piperacillin-tazobactam, are also advocated for their sake of larger distribution and less renal toxicity. Antibiotics must be changed as appropriate according to the culture results. In this study, most BNF patients were administered third-generation cephalosporins, meropenem or piperacillin-tazobactam by the judgement of each surgeon in charge. On the other hand, most OCNF patients were administered sulbactam/ampicillin and clindamycin, meropenem, or piperacillin-tazobactam. As the basic antibacterial therapy of OCNF, broad-spectrum of antibiotics and often more than one antibiotic including a penicillinase-resistant penicillin for streptococcal and staphylococcal bacteria, and aminoglycoside for Gram-negative bacteria, clindamycin or metronidazole for anaerobic organisms may be indicated [44, 45].

This study has some limitations. First, there is a possibility of unknown confounding factors because this was a retrospective study. Next, a few BNF patients were excluded because they transferred to other hospitals for various reasons during this study. If they were added to the BNF group, other results may have been obtained (e.g., mortality of BNF group may have been significantly higher than that of the OCNF group). Third, underlying diseases may affect the findings. Actually, univariate analysis of OCSI patients showed that "compromised host" was one of the significant factors of OCNF (Table 3). However, because OCNF is rare disease, we cannot compare two groups by matching the conditions (e.g., the number of compromised host). Although, we have to accept the results by considering the possibility of impact of underlying diseases. Finally, it is inevitable that the treatment content including antibacterial drugs will be involved in the prognosis (hospitalization periods and outcomes). Whether or not antibiotics was administered before hospitalization is also unknown. However, for emergency medicine, diagnosis is most important. We mainly focused on diagnosis of NF in this study. We hope that the results of this study will be useful for earlier diagnosis of OCNF by surgeons.

## 5. Conclusion

This is the first report to have investigated the characteristics of OCNF by comparing it with OCSC and BNF. We found that compared to BNF, OCNF can be detected at a lower clinical stage using indexes, including NLR and LRINEC score, besides clinical findings, which may contribute to patient’s relief.

In addition, we found that NLR may be useful for predicting OCNF. However, this is just an auxiliary diagnostic tool. Surgeons should comprehensively diagnose whether OCNF by adding clinical findings and image findings.
